# Crystalline and Electrical Property Improvement of Filtrated, Exfoliated Graphite Sheets by an In-Plane Current and Heating Treatment

**DOI:** 10.1186/s11671-020-03408-8

**Published:** 2020-10-02

**Authors:** Naoyuki Matsumoto, Azusa Oshima, Motoo Yumura, Kenji Hata, Don N. Futaba

**Affiliations:** National Institute of Advanced Industrial Science and Technology (AIST), Central 5, 1-1-1 Higashi, Tsukuba, Ibaraki, 305-8565 Japan

**Keywords:** Exfoliated graphite sheet, Current treatment, Heat treatment, Electrical conductivity, Defect healing

## Abstract

We report an approach to fabricate high conductivity graphite sheets based on a heat-and-current treatment of filtrated, exfoliated graphite flakes. This treatment combines heating (~ 900 °C) and in-plane electrical current flow (550 A·cm^−2^) to improve electrical conductivity through the reduction of crystalline defects. This process was shown to require only a 1-min treatment time, which resulted in a 2.1-fold increase in electrical conductivity (from 1088 ± 72 to 2275 ± 50 S·cm^−1^). Structural characterization by Raman spectroscopy and X-ray diffraction indicated that the improvement electrical conductivity originated from a 30-fold improvement in the crystallinity (Raman G/D ratio increase from 2.8 to 85.3) with no other observable structural transformations. Significantly, this treatment was found to act uniformly across a macroscopic (10 mm) sheet surface indicating it is on the development of applications, such as electrodes for energy generation and storage and electromagnetic shielding, as well as on the potential for the development of large-scale treatment technologies.

## Introduction

Advanced carbon materials possess advantages over many metals for use in sheets due to their flexibility, weight, and environmental resistance. These sheets (also called films) using carbon nanotubes or graphite have shown to be useful in a multitude of applications for flexible electronics, sensors, and electromagnetic shielding [1–11]. The high electrical conductivity is another area that has been investigated enabling increased performance in applications, such as radiofrequency, microwave passive components, and membranes [10–12]. Several groups have previously reported the fabrication of graphite-based sheet or films possessing excellent electrical conductivities in the range of 100–10,000 S·cm^−1^ using methods ranging from the exfoliation of graphite or the pyrolysis of polymers [12–20]. For example, Ohnishi et al. demonstrated the fabrication of graphite films directly through the pyrolysis of aromatic polymers at 3000 °C, which exhibited an electrical conductivity of 10,000 S·cm^−1^ [21]. In addition, Song et al. demonstrated the fabrication of graphite films for flexible radio frequency (RF) antennas by combining a high temperature treatment of polymer precursors and compressive rolling, which exhibited an electrical conductivity of 11,000 S·cm^−1^ [12]. In stark contrast, Behabtu et al. reported the fabrication of graphite sheets through the vacuum filtration of few layered graphene from exfoliated graphite powder, which showed an electrical conductivity of 1100 S·cm^−1^ [22]. Furthermore, Lotya et al. reported improvement of the electrical conductivity of exfoliated graphite thin film (thickness 30 nm) from 0.35 to 15 S·cm^−1^ by annealing the exfoliated sheet at 250 °C in Argon (Ar)/N_2_ for 2 h [23]. Wang et al. demonstrated that the electrical conductivity of the large-area, conductive and flexible reduced graphene oxide (RGO) membrane improved from 57.3 to 5510 S·cm^−1^ [24]. These examples demonstrate that, while solution-based processing represents an easier route of fabrication than the high temperature pyrolysis of aromatic polymers, the same level of electrical conductivity cannot be achieved. Interestingly, as demonstrated by several, particularly Song et al. [12] and Lotya et al. [23], a single-step process is insufficient to generate high electrically conducting sheets. This is analogous with the fabrication of carbon fibers which uses multiple steps including heating, strain, and carbonization to minimize the amount of crystalline defects to increase tensile strength from ~ 2 to ~ 10 GPa [25].

Motivated by these approaches, we report an approach to fabricate highly electrically conducting graphite films by simple exfoliation and a heat-and-current treatment. Using graphite sheets made by vacuum filtration, the treatment involves simultaneous heating in a neutral gas ambient combined with an in-plane electrical current flow. Requiring only a 1-min treatment time, a two-fold improvement in the electrical conductivity to 2275 ± 50 S·cm^−1^ could be achieved. Structural analysis of the treated graphene sheets showed a 30-fold improvement in crystallinity (as determined by Raman spectroscopy) which correlated well with the observed conductivity increase.

## Methods/Experimental

### Graphite Exfoliation and Sheet Preparation

**Fig. 1:**
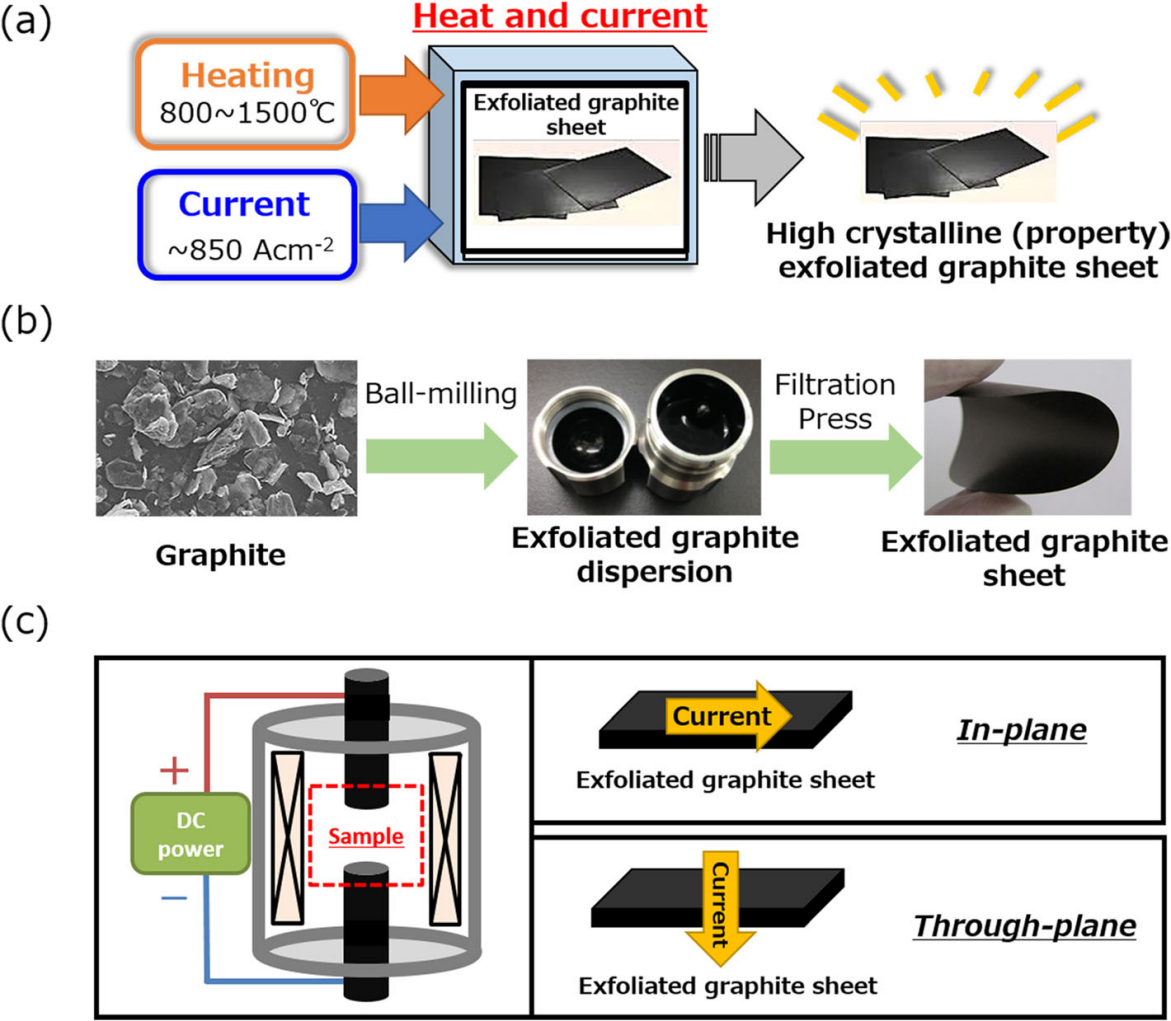
**a** Conceptual figure illustrating the heat and current post-treatment for filtrated, exfoliated graphite sheets. **b** Preparation process of the exfoliated graphite sheet from as-purchased graphite flakes to an exfoliated graphite dispersion and to sheet. **c** Primary components (chamber, heater, and electrodes) of the treatment apparatus (left) and the two configurations for passing current in-plane or through-plane (right)

Graphite sheets were prepared through filtration of a dispersion of exfoliated graphite powder. Commercially available highly purified graphite powder (ACB-100) was purchased from Nippon Graphite Industries, Co., Ltd., which consisted of ~ 80 μm-sized particles with thickness of 500–1000 nm. Two milligrams of this graphite powder was mixed with 50 mg of dodecyl benzene sulfonic acid (Tokyo Chemical Industry Co., Ltd.) as a dispersant in 10 ml of hydro-fluoroether (C_4_F_9_OC_2_H_5_, Novec 7200 in 3.0 M, surface tension; 13.6 mN·m^−1^). Exfoliation was performed by ball-milling (Verder Scientific Co., Ltd.) using stainless steel ball-bearings for 30 min at 10 Hz. Following the ball-mill exfoliation, the size and thickness of the graphite flakes was reduced to ~ 500 nm and ~ 45 nm, respectively by atomic force microscopy (AFM) (supplemental Fig. 1a). Therefore, on average, the dispersed flakes contained ~ 130 graphene. The dispersion was vacuum filtrated to form free-standing sheets. Following the filtration, exfoliated graphite sheets possessed thicknesses between 27 to 48 μm (average 35 μm) as characterized by a thickness gauge (Dektak XT, Bruker). The SEM image, Raman spectra, and XPS spectra of this exfoliated graphite sheet are shown in supplemental Fig. 1b-d. These sheets were rinsed in distilled water to remove residual chemicals and then dried at 100 °C in air for 24 h [26]. Finally, the sheets were subjected to uniaxial pressure (~ 0.5 MPa) to increase packing density and electrical conductivity (Fig. 1b).

### Equipment and Process for Heat and Current Technique

In general, the processing equipment for simultaneous heating and current flow is composed of three parts: (1) a chamber suitable for low vacuum to atmospheric pressures to control the ambient, (2) a high-frequency induction heating system (up to 2000 °C), and (3) opposing circular electrodes (10-mm diameter) composed of isotropic graphite to apply high current (maximum 266 A at 120 V, or ~ 850 A·cm^−2^ DC). The system is described in detail in a previous report [27, 28]. In this series of experiments, an Ar ambient was used.

Important to this current examination was the design of the opposing co-planar electrode contact surfaces to accommodate both in-plane and through-plane current flow (Fig. 1c). For the through-plane current configuration, the sheet was sandwiched between the two opposing electrode surfaces as illustrated in Fig. 1c. For the in-plane current configuration, as illustrated in supplemental Fig. 2, custom-shaped electrodes were prepared with a rectangular recess to allow the insertion of an electrically insulating zirconia plate. In this way, the sheet is sandwiched between the two custom contacts, but the current flow passes in the plane of the sample. To confirm stability and reproducibility of the treatment, each experiment was performed five times, and the average values and standard deviations are reported. We would like to note that this treatment is not an in situ CVD process as source precursors are not introduced to initiate growth. This process only supplies energy, through a combination of heat and electrical current, to induce the healing of defects in the graphene sheets.

The standard treatment time was chosen to be 1 min because our results of the time-dependent effects of the treatment revealed that only nominal improvement was observed for a 30 s time, and for over 1.5 min, damage to the graphene structure was observed. The damage was evidenced by a decrease in the G/D ratio as shown in Supplemental Fig. 3a. Using the 1-min treatment time, the standard treatment temperature was determined by investigating the temperature dependence on the electrical conductivity in the range of 800 to 1000 ^°^C (Supplemental Fig. 3b).

### Characterizations

The surface resistance of the exfoliated graphite films was performed using a four-probe electrical measurement tester (Loresta GP MCP-T610, Mitsubishi Chemical Analytech Co., Ltd.).

Structural characterization was examined with X-ray diffraction (XRD) Cu Kα (λ = 0.15418 nm, MiniFlex II, Rigaku Corporation). The interlayer distance was estimated using Bragg’s diffraction formulation (1);

1 $$ \lambda =2\kern0.5em d\cdot \sin \kern0.5em \uptheta $$

where *d* is the interlayer distance (theoretical interlayer distance of graphite is 0.335 nm).

The Raman graphitic to disorder intensity ratios (G/D ratio) of the exfoliated graphite films before and after the heat and current treatment were examined using a Raman Spectrometer (XploRA, HORIBA, Ltd.) at an excitation wavelength of 532 nm (sampling area 100 μm). After baseline correction of the obtained Raman spectra, each peak intensity of 1300 to 1400 cm^−1^ (D-band) and 1580 to 1620 cm^−1^ (G-band) was measured. In addition, 2D peak location and intensity were observed relative to the G-band. To provide an overall and accurate sampling of the sheet, Raman measurements were performed in 10 positions distributed throughout the graphite films, and each band intensity of Raman G/D ratio was calculated and averaged. The detailed measurement conditions were as follows: Spectroscope: Czerny Turner type detector with 200 mm of focal length, resolution (slit width of 100 μm): 2–15 cm^−1^, and laser output 20–25 mW.

## Results and Discussion

We begin our two-step process by characterizing the electrical conductivity of the vacuum-filtrated graphite sheets. As described in the “Methods/Experimental” section, the electrical conductivity of the ~ 35-μm thick (average) sheets was performed using a four-probe electrical resistivity measurement device. The average electrical conductivity was found to be 1088 ± 72 S·cm^−1^ which compares well with other filtrated graphite and graphene sheets.

**Fig. 2:**
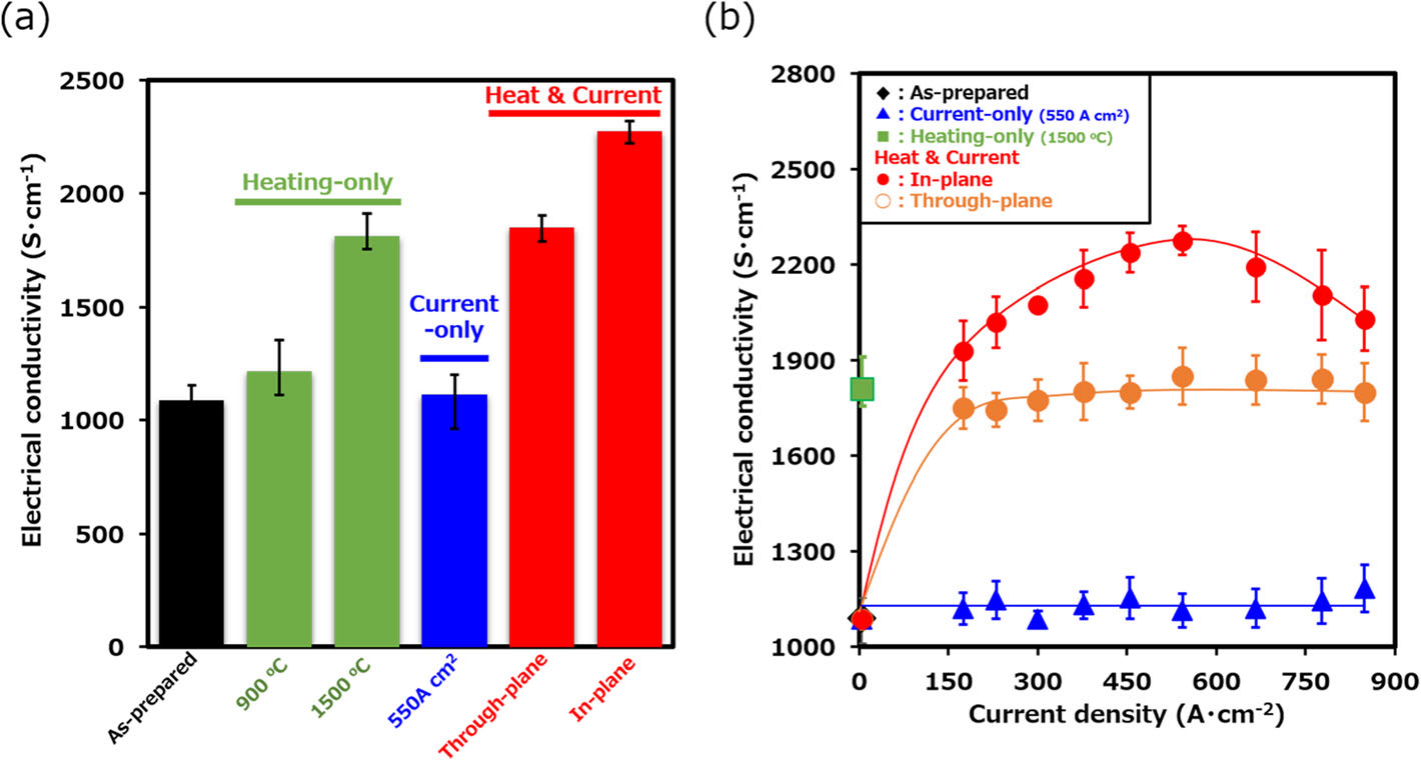
**a** Electrical conductivity of the graphite sheets for the as-treated case and following different treatment conditions. **b** Dependence of the electrical conductivity dependence on the applied current density for different treatment conditions (Current-only, heat and in-plane current, and heat and through-plane current.) Heating-only and as-prepared are included as reference.

These sheets were then subjected to the heat-and-current treatment to increase electrical conductivity. Our results demonstrate the necessity and advantage of simultaneous heating and in-plane current flow on the enhancement of the graphite sheet properties. We applied a combined treatment of current flow and heating as previously reported for single wall carbon nanotubes (SWCNTs) [27, 28]. Using a heating temperature of 900 °C, we investigated the dependence of the treated sheet electrical conductivity as a function of the applied in-plane current density. For each point, the temperature was raised to 900 °C, and in-plane current (0 to 850 A·cm^−2^) was passed for 1 min. The electrical conductivity of each sheet was measured and plotted (Fig. 2b). The plot of the sheet electrical conductivity versus applied current density exhibited a sharp increase from the as-prepared value (1088 ± 72 S·cm^−1^) to as high as 2250 ± 50 S·cm^−1^ at 550 A·cm^−2^, which was followed by a decrease at elevated current densities (850 A·cm^−2^) (Fig. 2b). Based on the decrease in the Raman G/D ratios (85.3 ± 5.7 at 550 A·cm^−2^ to 10.7 ± 1.0 at 850 A·cm^−2^), we suspect that the observed decrease in the electrical conductivity at current densities beyond ~ 550 A·cm^−2^ is a result of a structural degradation through mechanisms, such as electro-migration. Based on these results, the optimum treatment condition for an Ar gas ambient was determined to be 550 A·cm^−2^ at 900 °C. These results demonstrate the effectiveness and advantage of the simultaneous use of heat and current flow.

To verify the importance of the combined heating and in-plane electrical current flow treatment, we conducted several control experiments using a (1) heating-only treatment, (2) a current-only treatment, and (3) a combined heating and through-plane current flow. In short, no other process condition demonstrated the equivalent level of electrical conductivity improvement as described above. First, the heating-only treatment was carried out at 900 and 1500 °C in an Ar ambient for 5 h. Similar to the results observed for CNTs, heating-only showed observable improvement at temperatures at, or above, 1500 °C [29]. As summarized in Fig. 2a (green), treatment at 900 °C resulted in a nominal improvement in electrical conductivity (1215 ± 70 S·cm^−1^), and treatment at 1500 °C resulted in a more significant increase to ~ 1812 ± 79 S·cm^−1^. In addition, in both cases, we observed a slight weight (~ 3%) decrease, which is likely due to the removal, perhaps degassing, of residual chemicals remaining from the exfoliation process. These results show that heating alone can be effective in improving the electrical conductivity of graphite sheets, but it requires temperatures exceeding 1500 °C and requires treatment time of hours.

Second, we examined the effect of a current-only treatment. In this test, electrical current was flowed in-plane for several samples ranging from 175 to 850 A·cm^−2^ for a 1-min treatment time. Following the treatment, the sheets exhibited no noticeable improvement in the electrical conductivity (blue bar in Fig. 2a, triangle in Fig. 2b). This result indicates the ineffectiveness of this current-only treatment. We suspect that the ohmic heating caused by the current is insufficient to induce any significant change to the sheet crystallinity and electrical conductivity.

Third, to demonstrate the importance of the current flow direction (through-plane versus in-plane) in the combined heat and current process, the current flow was passed through-plane on a series of graphite sheets using the electrodes without the insulating alumina plates as described in the “Methods/Experimental” section. The graphite sheets were subjected to current densities spanning a range of 175 to 850 A·cm^−2^, and the electrical conductivity was measured and plotted as a function of the applied current (Fig. 2b). From this plot, we make several observations. One, the effect on the electrical conductivity is immediate with relatively low applied currents. With the lowest applied current density (150 A·cm^−2^), the electrical conductivity of the graphite sheet increased about 70%. Second, further increase in applied current resulted in no further improvement. Third, the level of electrical conductivity increase (~ 1812 ± 79 S·cm^−1^) was equivalent to the results of the heating-only examination, but in contrast, only required a 1-min treatment time.

Taken together, these results indicate the synergetic effect of a combined heating and current treatment. Application of a 1-min treatment at 900 °C heating combined with through-plane current improved the level of electrical conductivity similar to that of the heating-only treatment (1500 °C, 5 h). However, additional improvement was not observed with increased applied current, which suggests that under the conditions of the heating and through-plane current, the energy provided is insufficient to induce further change to the graphitic structure. We suspect that the through-plane current flow induces ohmic heating which essentially reduces this arrangement equivalent to a heat-only treatment. In addition, the weak dependence of the electrical conductivity on the applied current indicates that the mechanism driving the improvement is not solely a thermal process (Fig. 2b). The possibility remains that the treatment time is too short despite the increased temperature. This hypothesis would explain the observed weak dependence on the applied current. Therefore, these results indicate the importance of combining heating with an in-plane current flow to achieve an effective and efficient treatment process for improving electrical conductivity of the filtrated, graphite sheets.

**Fig. 3:**
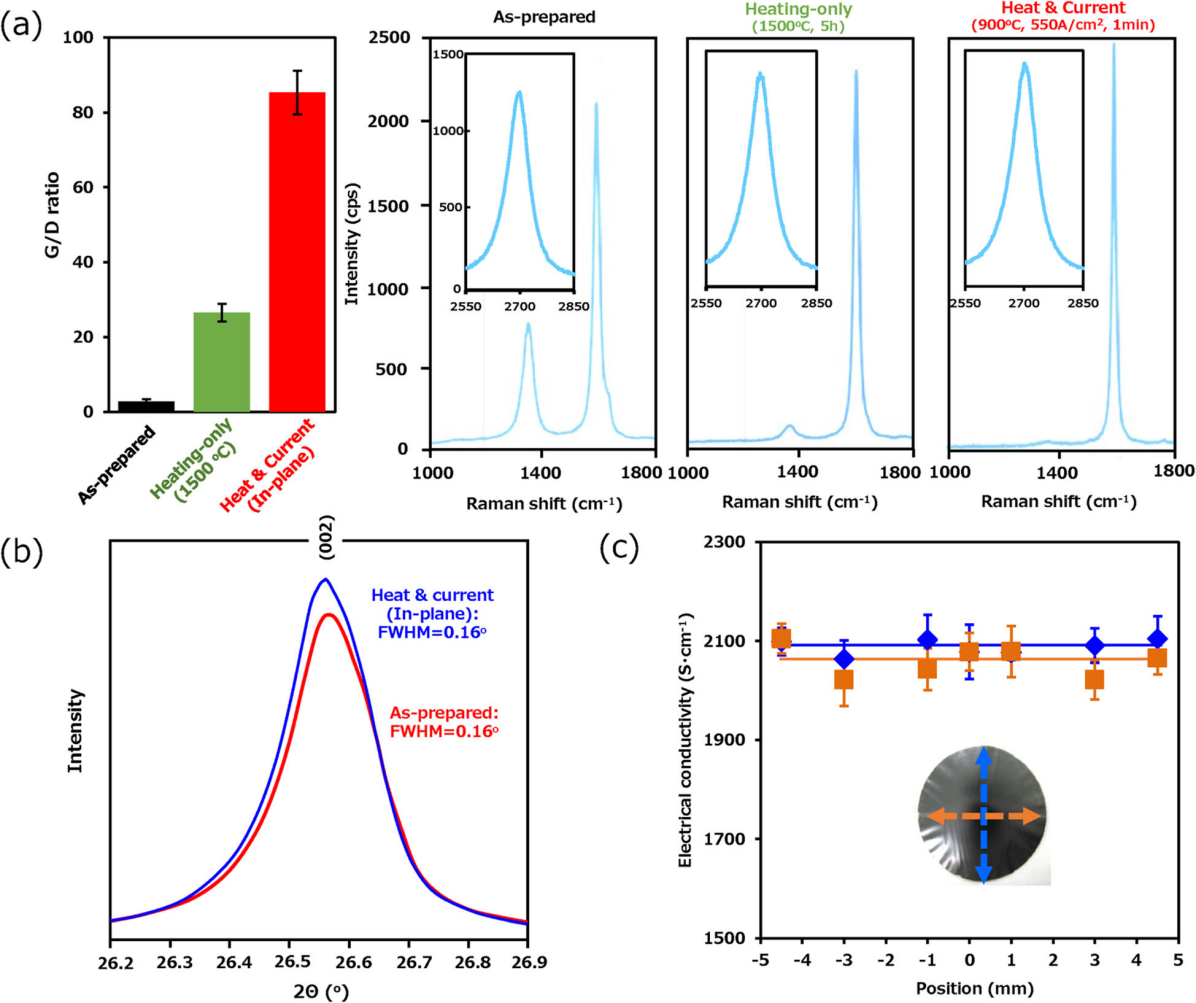
**a** Raman spectra of the filtrated, exfoliated graphite sheets before and after heat and current process and after the heating-only process. **b** FWHM of XRD (002) reflection of as-prepared and processed exfoliated graphite sheet. **c** Uniformity of the electrical conductivity across the processed exfoliated graphite sheet surface

As our treatment acts onto a macroscopic assembly of graphite flakes filtered into a 10 mm × 10 mm sheet, homogeneity in the improvement is critically important. Previous reports using DC/AC current and plasma treatments have shown difficulty in uniformly processing the entire surface [30]. Large treatment variation is an obstacle in future scale-up development as well as application development. To address this point, the electrical conductivity uniformity was evaluated at 0, ± 1.0, ± 3.0, and ± 5.0 mm from center of the treated graphite sheet (φ10 mm). As seen in Fig. 3c, the mean electrical conductivity was ~ 2275 ± 50 S·cm^−1^ with a variance of only 1.5% (x, ~ 0.7 %; y, ~ 1.5%). This result demonstrates that the heat and current treatment acted on the entire graphite sheet exceptionally uniformly and suggests the possibility of future endeavors of scale up.

To attempt to clarify the origin of the improved electrical conductivity, structural characterization of the sheets before and after treatment was performed. Crystallinity characterization was carried out using Macro-Raman spectroscopy sampled across the graphite sheet surface as described in the “Methods/Experimental” section. Taken together, the spectra for the graphite films before and after treatment showed the characteristic features of graphite: a sharp graphitic band, a disorder band, and a 2D band. The Raman G/D ratio of the heat- and current-treated sheet increased over 30-fold to ∼ 85.3 ± 5.74 from that of the as-prepared sheets (G/D ratio ~ 2.8 ± 0.55). A previous report by Jin et al. reported that the defects in SWCNTs migrate through the passage of current along nanotubes [31]. As such, we hypothesize that the treatment induces defect migration toward the edges of the graphite domains. This may explain the need for the in-plane current flow. For comparison, sheets treated by heating-only at temperatures of 1500 °C exhibited a 9.5-fold increase in G/D ratio (~ 26.5 ± 2.38) (Fig. 3a). The G/D ratio of the sheets treated by current-only treatment was found to be 2.7 ± 1.96 ranging from 175 to 850 A·cm^−2^, which indicated no enhancement from the applied current density (not show these spectra) similar to the electrical conductivity in Fig. 2b. This result shows that the crystallinity improvement correlates well to the improved electrical conductivity. The 2D peaks were observed for all samples at ~ 2700 cm^−1^ before and after heat and current process. The similarity in the peak position indicates that the layer number was not obviously changed by the heat and current process [32].

Structural characterization by X-ray diffraction (XRD) was carried out on the untreated and heat- and current-treated sheets (Cu Kα: *λ* = 0.15418 nm, MiniFlex II, Rigaku Corporation). Observation of the (002) reflection at 2θ = 26.5° revealed no obvious change in the reflection position and profile shape as a result of the treatment (Fig. 3b). This means that the interlayer distance of the treated and untreated sheets, which was estimated to be ~ 0.335 nm, was unaffected by the treatment. In addition, the full width half maximum (FWHM) of the (002) reflection, which is related to the layer spacing, was also unaffected at ~ 0.16°. These results suggest that the observed improvement to the electrical conductivity does not arise from an improvement in the interlayer spacing of the individual flakes. Taken together, the Raman and XRD results suggest that the improvement in crystallinity, as well as related structural features, such as inter-particle junctions or merging of adjacent domains, appears to be the primary origin of the observed increase in electrical conductivity. Our attempts at microscopically observing this phenomenon were unsuccessful.

We wish to comment on the limitations and the possibility of scale-up of this process. While this treatment shows potential in the effective and efficient property improvement of graphite sheets, we do recognize the need for a high-power source as well as relatively high treatment temperatures (~ 900 °C) to remain time efficient. Based on our previous work on the treatment of single-wall carbon nanotubes, the treatment temperature can be decreased with an associated increase in the treatment current [29]. Therefore, one possible approach to reduce the temperature to ~ 800 °C would be to increase the applied current ~ 20%. In addition, previous studies have shown that this process is fundamentally scalable by treating multiple sheets simultaneously and obtaining similar results. Given that this process can efficiently improve the electrical conductivity (from 1088 ± 72 to 2275 ± 50 S·cm^−1^) of exfoliated graphite in only 1 min, we envision that this technique should be suitable for a roll-to-roll process to allow for continuous and large-scale treatment of graphite sheets. Therefore, this work can have significant implication in the improvement of macroscopic and highly conductive graphite films for electrode materials for nanocomposites with electrical conductivity, electromagnetic shielding, and photonic devices.

## Conclusions

In conclusion, we have demonstrated an approach to fabricate highly conducting graphite sheets consisting of exfoliated graphite sheets and a treatment consisting of a combined heating and in-plane electrical current flow. This treatment was found to be critical in improving the electrical conductivity of exfoliated graphite sheets 2.1-fold from 1088 ± 72 to 2275 ± 50 S·cm^−1^. We do note that our level of electrical conductivity remains about 20% of that reported by Song et al. (11,000 S·cm^−1^, 5–8 h) [14], but in contrast, this approach requires only a 1-min treatment time. Further, our approach uniformly treated the entire 10-mm sheet within a variance of 1.5%, which has significant implications for both the application of this material as well as the possibility for scale-up.

## Supplementary information

**Additional file 1:.** Supplemental Fig. 1 Characterizations of exfoliated graphite dispersion and sheet. (a) AFM image, and (b) thickness and size histogram of exfoliated graphite dispersion, and (c) XPS spectra, and (d) SEM image of exfoliated graphite sheet. Supplemental Fig.2 Equipment diagram of Heat and current process for filtrated, exfoliated graphite sheets. Supplemental Fig. 3 Relationship between the electrical conductivity of exfoliated graphite sheet and (b) treatment time and (c) treatment temperature.

## Data Availability

All data are fully available without restriction.

## Abbreviations

CVD: Chemical vapor deposition
N_2_: Nitrogen
Ar: Argon
XRD: X-ray diffraction
DC: Direct-current
AC: Alternating current
FWHM: Full width half maximum
AFM: Atomic force microscope
