# Supplementary material for: Crystalline and Electrical Property Improvement of Filtrated, Exfoliated Graphite Sheets by an In-Plane Current and Heating Treatment
Source: Nanoscale Res Lett. 2020 Oct 2;15:195. doi: 10.1186/s11671-020-03408-8 (PMC7532253; doi:10.1186/s11671-020-03408-8)
Supplement: Supplementary file 1 — Additional file 1:. Supplemental Fig. 1 Characterizations of exfoliated graphite dispersion and sheet. (a) AFM image, and (b) thickness and size histogram of exfoliated graphite dispersion, and (c) XPS spectra, and (d) SEM image of exfoliated graphite sheet. Supplemental Fig.2 Equipment diagram of Heat and current process for filtrated, exfoliated graphite sheets. Supplemental Fig. 3 Relationship between the electrical conductivity of exfoliated graphite sheet and (b) treatment time and (c) treatment temperature. [file 11671_2020_3408_MOESM1_ESM.pdf]

Dear Warjun S. Dagunton,

We are very sorry.

The file updated to your journal was locked for in-house viewing.
We attached the unlocked file.

Thank you for your continued editing work.

Best Regards,
Naoyuki Matsumoto
